# Liver Disease Secondary to Intestinal Failure

**DOI:** 10.1155/2014/968357

**Published:** 2014-01-15

**Authors:** Bassam Abu-Wasel, Michele Molinari

**Affiliations:** Queen Elizabeth II Health Sciences Centre, Dalhousie University, 1276 South Park Street, Office 6-302 Victoria Building, Halifax, NS, Canada B3H 2Y9

## Abstract

IFALD is a common and potentially life-threatening condition for patients with SBS requiring long-term PN. There exists the potential for decreasing its incidence by optimizing the composition and the rate of infusion of parenteral solutions, by advocating a multidisciplinary approach, and by early referral for intestinal-liver transplantation to ensure long-term survival of patients with SBS.

## 1. Introduction

Intestinal failure (IF) refers to any clinical condition that alters the physiological absorption of nutrients. It presents with the inability to maintain the necessary caloric balance and the homeostasis of proteins, carbohydrates, lipids, fluids, electrolytes, and other micronutrients [1].

The most common causes of IF are extensive surgical intestinal resections, chronic intestinal obstruction, dysmotility disorders, and congenital defects. The short bowel syndrome (SBS) is a type of IF caused by intestinal resections leading to less than 200 cm of residual bowel [2, 3]. Patients with SBS are at risk of severe metabolic, renal, and hepatobiliary complications in addition to central venous catheter infections and venous thrombosis. The introduction of parenteral nutrition (PN) in the 1960s changed the prognosis of patients with SBS [4]. Although PN is necessary to sustain patients with IF, it is associated with several side effects and liver injury is one of the most prevalent and severe complications. Liver decompensation induced by long-term use of PN is referred to as intestinal failure associated liver disease (IFALD).

## 2. Epidemiology and Etiology of SBS

The incidence and prevalence of SBS are estimated to be 3 and 4 per million individuals, respectively [5]. In the adult population, SBS accounts for approximately three-fourths of intestinal failures and more than one half in children. The majority of patients suffering from SBS underwent an extended resection while only a fourth underwent multiple sequential intestinal resections such as in patients with Crohn's disease [6]. During the last decades, there has been a significant improvement of the life expectancy of these patients primarily due to the introduction of PN. However, massive intestinal resection continues to be associated with significant morbidity and mortality [6, 7] and only 70% of patients are able to leave the hospital [7] and their expected 5-year survival is 75% [8].

The causes of SBS are different between the adult and the pediatric population [9]. In adults, superior mesenteric artery insufficiency, superior mesenteric vein thrombosis, small bowel volvulus, strangulation, congenital malrotation, and recurrent strictures or fistulas due to Crohn's disease and radiation enteritis are the most common causes [10–16] (Table 1).

In infants, necrotizing enterocolitis is the most common etiology of SBS, followed by intestinal atresia, gastroschisis, and midgut volvulus [17].

## 3. Pathophysiology of Short Bowel Syndrome

The absorptive capacity of the intestinal tract is far in excess of need. The length of the small bowel is between 3 and 8 meters, while the colon is 1 to 2 meters [18, 19]. Most of the absorption and digestion of carbohydrates and proteins take place in the duodenum and proximal jejunum. The ileum is responsible for absorbing bile salts, fat-soluble vitamins, and vitamin B12 bound to intrinsic factor. Fluids and electrolytes are absorbed mainly in the ileum and the large bowel [20, 21]. The degree of functional intestinal impairment is dependent on a number of factors such as the overall length of the intestine, what segments of the bowel are preserved, the absorptive quality of the remnant bowel, and the interindividual variability for the efficiency of the absorptive capacity.

## 4. Quantitative Loss of Absorptive Capacity

The small bowel has a large functional reserve capacity. Thus, resection of up to 50% of the small bowel is usually tolerated without any symptoms. In most patients, resection of up to 50–70% leads to transient malabsorption, and if the intestinal residual length is less than 60–100 cm, almost all patients will require long-term PN. Patients with extensive small bowel resections but with functional colons are able to adjust better to the situation as the residual large intestine adopts important digestive functions and maintains the necessary absorption of water and electrolytes.

## 5. Qualitative Loss of Absorptive Capacity

Losses of the duodenum or the terminal ileum, in particular the ileocecal valve, impair the absorption of nutrients much more than the loss of other parts of the small bowel. The duodenum plays an important role in the stimulation of digestive responses, in particular of pancreatic enzyme output. Duodenal resection leads to significant maldigestion of nutrients which further impairs the intestinal absorption. In healthy humans, exposure to nutrients at the terminal ileum induces inhibition of digestive secretory and motor functions, the so-called ileal brake [22–24]. The lack of the “ileal brake” causes gastric hypersecretion and accelerated small bowel transit with malabsorptive diarrhea.

## 6. Carbohydrate and Protein Malabsorption

About 70% of the caloric intake from carbohydrates is reabsorbed in the presence of 60–100 cm of small bowel. Carbohydrate malabsorption is of limited importance in patients with normal colon because up to 80% of carbohydrates not absorbed by the small bowel can be absorbed after bacterial metabolism to short chain fatty acids in the colon [25]. On the other hand, the absorption of other food components varies [26, 27].

## 7. Calcium, Magnesium, Iron, and Vitamins Malabsorption

Calcium, magnesium, iron, and folic acid are predominantly absorbed by the duodenum. Malabsorption of calcium may be particularly severe because absorption is further hampered by binding of calcium to malabsorbed fatty acids and by vitamin D deficiency. In healthy subjects, calcium binds to oxalate to form unabsorbable calcium oxalate. In SBS patients, lack of free intraluminal calcium allows increased absorption of unbound oxalate. About 60% of patients with SBS develop hyperoxaluria and are at an increased risk of calcium oxalate kidney stones.

## 8. Fat and Fat Soluble Vitamins Malabsorption

Fat malabsorption leads to steatorrhoea and malnutrition and is associated with deficiencies of the fat soluble vitamins A, D, E, and K. On the other hand, vitamins B1, B2, B6, and C are absorbed along the entire small bowel, and deficiencies of these vitamins are relatively rare. Resection of the ileum leads to vitamin B12 deficiency and spill-over of unabsorbed bile acids into the colon, which causes choleretic diarrhea. Lack of trace elements, in particular zinc and selenium, occurs in patients with SBS and results in epithelial and mesenchymal dysfunction as well as immunodeficiency with subsequent increased risks of severe infections [28].

## 9. Bile Acid Malabsorption

After resections of more than 60–100 cm of the ileum, the enteric loss of bile acids usually exceeds the synthetic function. The physiologic bile acid pool declines and the risk of cholesterol gall stones and steatorrhoea increases significantly. Excessive colonic levels of bile salts solubilise unconjugated bilirubin and promote its absorption. This leads to 3–10-fold increase in the concentration of bilirubin in the bile of patients with ileal resections [29, 30] and predisposes to the formation of pigment gallstones.

## 10. Adaptation

Extensive intestinal resections are followed by 3 phases of adaptation [2, 31].

1. *The Acute Phase.* Starts after the intestinal resection and lasts for a period of 4 weeks when the intestine mucosa and peristalsis begin to readjust to the new environment.
2. *The Adaptation Phase.* Lasts for 1 to 2 years. Patients usually require PN in combination with enteral feeding, until their bowel has undergone sufficient remodeling. Intestinal failure is considered permanent when specialized nutritional support is required beyond this initial 2-year period.
3. *The Maintenance Phase.* During this phase permanent nutrition treatment should be individualized according to the extent and quality of nutritional deficits.

**Figure 1:**
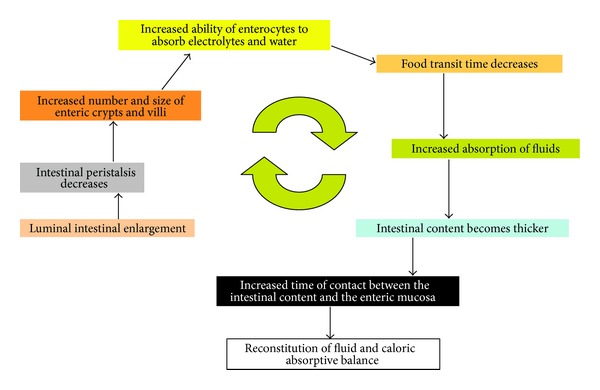
Schematic representation of the adaptive mechanisms of the intestine after extensive resections.

Adaptation of the intestine is mainly stimulated by exposure of the residual mucosa to macronutrients [32, 33]. The bowel increases in both length and diameter. This is accompanied by hyperplasia of the small intestinal mucosa with increased number and size of crypts and villi. Individual cells increase certain absorptive functions [34, 35] and the intestinal motility slows down. The time nutrients are in contact with the intestinal mucosa increases significantly leading to better absorption of the intestinal content (Figure 1).

## 11. Liver Disease Associated with Intestinal Failure 

SBS severity depends on the intestine length and its adaptive capacity [36]. The introduction of PN has given rise to a new hope in the treatment of IF associated with the SBS providing an increase in survival of these patients. One of the most prevalent and severe complications in SBS patients on PN is hepatobiliary dysfunction, commonly referred to as intestinal failure-associated liver disease (IFALD). IFALD is defined as the persistent elevation of serum transaminases 1.5 times above the upper limit of normal in the presence of SBS [37].

It is often difficult to determine the degree to which hepatocellular dysfunction is a consequence of the SBS, nutritional support, or drug therapy used for the management of SBS.

Three types of hepatobiliary disorders are associated with IFALD:

- steatosis,
- cholestasis,
- formation of gallbladder stones or sludge.

Steatosis is more prominent in adults, while cholestasis is more prominent in children and both can progress to fibrosis, cirrhosis, and end-stage liver disease.

The full spectrum of IFALD is summarized in Table 2.

## 12. Etiology of IFALD

The etiology of IFALD is complex and still poorly understood (Table 3). About 25% to 100% of adult and children requiring PN have elevated liver function tests [17]. Prospective studies have shown that the prevalence of complicated IFALD increases with longer duration of PN. Chronic cholestasis occurred in 65% after a median of 6 months (range, 3–132 months). Complicated liver disease was observed in 37% after a median of 17 months (range, 2–155 months). The prevalence of complicated IFALD was 26% ± 9% at 2 years and 50% ± 13% at 6 years. Among all the causes of death, 22% of patients expired for end-stage liver disease [47]. In another study of 42 adults receiving home PN for more than 1 year in the USA, 6 (15%) developed end-stage liver disease. All 6 patients died, at an average of 10.8 ± 7.1 months after they experienced cholestasis [48].

**Figure 2:**
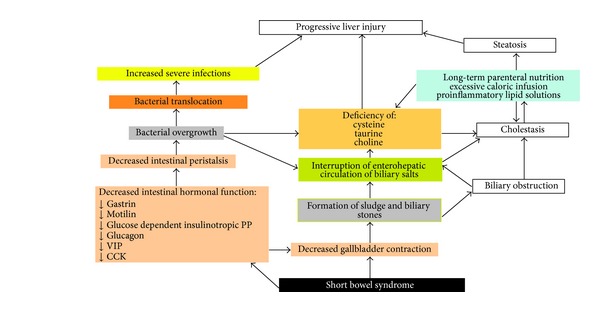
Schematic representation of the most common causes of liver injury in patients with short bowel syndrome on long-term parenteral nutrition.

The interruption of the normal enterohepatic circulation of biliary salts causes an abnormal metabolism of the biliary acids (Figure 2). This predisposes to bacterial translocation and systemic sepsis. Parenteral lipid intake greater than 1 g/kg/day is a predisposing factor for cholestatic liver disease that may be caused by the activation of macrophages induced by the excess of omega-6 polyunsaturated fatty acids [49]. In addition, there is recent evidence of manganese toxicity in patients on prolonged PN. Because manganese is excreted in the bile, its toxic effect is exacerbated in cholestasis [50, 51].

Gastrointestinal hormones levels such as gastrin, motilin, glucose-dependent insulinotropic polypeptides, secretin, pancreatic polypeptide, glucagon, and vasoactive intestinal peptides are reduced in patients who are dependent on PN. This leads to the decrease of gallbladder contractibility and intestinal stasis. Intestinal stasis is responsible for bacterial overgrowth, translocation, recurrent infections, and the production of lithocholic acid that has been shown to be hepatotoxic in rats [36, 49].

In addition, the lack of cholecystokinin (CCK) reduces the peristalsis of the gallbladder and predisposes to the formation of biliary sludge [49] within a few weeks of initiating PN in the complete absence of enteral nutrition. The incidence of biliary sludge increases with the duration of PN and it has been diagnosed in 6% of patients within 3 weeks and in 100% within 6 to 13 weeks of PN [49]. Cholelithiasis occurs in 20% to 40% of both adult and pediatric patients on PN [17, 52–54]. Factors that predispose to gallstone formation include the abnormal metabolism and secretion of bile, gallbladder stasis, and malabsorption of bile acids. Depending on the dominant mechanism, either mixed pigment stones or cholesterol stones may occur in the biliary system of these patients. The risk for cholelithiasis is significantly increased if less than 120 cm of intestine is preserved, if the terminal ileum has been resected, and if long-term PN is required. In these circumstances, the gallbladder becomes atonic and the enterohepatic cycle of the bile acids is interrupted inducing lithogenic bile and cholesterol stones. Although the presence of the colon reduces the fluid losses, colon preservation does not affect the incidence of cholelithiasis in patients on long-term PN [55].

IFALD is also caused by either deficiency or excess of amino acids and lipids in PN solutions. Taurine and cysteine deficiency is associated with hepatotoxicity in neonates. Choline deficiency may exacerbate hepatic steatosis both in children and adults. In a pilot study, the addition of 2 gm of choline chloride reduced steatosis in adults, with the normalization of hepatic transaminases and resolution of abnormal finding of the liver by computed tomography [49]. The excess of lipidic calories leads to hepatic steatosis, hyperlipidemia, and thrombocytopenia. Kelly showed that cholestasis in adults is related to usage of more than 1 g/kg/day of lipids, and the damage mechanism is the direct effect of lipids in the hepatocytes, accumulation of phospholipids or phytosterols, or the production of inflammatory cytokines [49].

## 13. Evaluation of Patients with Liver Dysfunction on Parenteral Nutrition

Adult patients with elevated liver enzymes are generally asymptomatic or affected by nonspecific symptoms such as fatigue, malaise, and right upper quadrant discomfort. Symptoms and signs of advanced IFALD are similar to those seen in other liver diseases and include jaundice, hepatomegaly, splenomegaly, ascites, edema, spider nevi, palmar erythema, and caput medusae.

The clinical evaluation of patients with SBS and suspected IFALD requires the exclusion of other causes that could be responsible for the abnormal liver function tests. A careful review of all the potential hepatotoxic medications and herbal supplements is mandatory. Clinicians taking care of these patients should also have a low threshold to order radiological investigations to rule out the presence of biliary obstruction and serological tests to rule out the presence of hepatitis and other types of infections that can induce cholestasis.

Serial assessment of biochemical tests is recommended including conjugated and direct bilirubin and g-glutamyl transferase to assess for cholestasis, albumin, and international normalized ratio for the hepatic synthetic function, alanine aminotransferase for possible hepatic injury, and platelet count and absolute neutrophil count for possible signs of portal hypertension. The most frequent laboratory hepatic abnormalities observed in patients on long-term PN are elevations in serum levels of lactate dehydrogenase, alkaline phosphatase, and total bilirubin. Abnormal laboratory values are predictive of the progression of liver disease.

Despite the utility of serial blood work, the gold standard for diagnosis of IFALD is definitive histopathology from a liver biopsy specimen [49]. Pathologic review of liver specimens shows a clear progression of disease starting with vesicular deposit of lipids in hepatocytes with displacement of cellular structures. When the intracellular lipid content reaches a critical level, inflammatory mediators are responsible for the release of oxygen radicals causing mitochondrial and cellular membrane dysfunction resulting in a chronic proinflammatory environment. In the proinflammatory process which is not reversed, periportal inflammation, bile duct proliferation and cholestasis, and bridging fibrosis and cirrhosis result in a permanent damage to the liver of these patients [38, 42, 45, 56] (Table 4).

## 14. Prevention and Treatment of IFALD

### 14.1. Maximizing Oral or Enteral Nutrition

Oral and enteral nutrition are known to reverse intestinal mucosal hypoplasia and preserve the immunologic integrity of the gut [57]. In addition, oral and enteral nutrition stimulate motility and reduce intestinal stasis, bacterial translocation, and the production of cytokine and endotoxins. Additionally, oral and enteral nutrition stimulate cholecystokinin (CCK) secretion and stimulate gallbladder contraction reducing the risk of gallstone and sludge formation [58].

### 14.2. Optimizing Parenteral Nutrition

During the last decades, several factors related to the rate of infusion, total daily caloric intake, and composition of PN have been identified as potential causes of IFALD. For example, glucose infusion, at a rate of more than 5 mg·kg^−1^·min^−1^, and lipid infusion at a rate of more than 1 g·kg^−1^·/d^−1^ are associated with the risk of overfeeding causing steatosis and cholestasis in adult patients [47, 59]. Therefore, precise individual caloric requirements are important to avoid these issues. This can be obtained by using the Harris Benedict equation or other validated methods to estimate the caloric needs for each patient. It is also important to maintain a balance in macronutrient composition in the TPN prescription. Hepatic steatosis occurs in over 50% of patients receiving dextrose infusion, as compared with 17% receiving a combined dextrose-lipid mixture [60]. In adults, it is recommended to provide glucose and lipids in a proportion of about 70% : 30% of nonprotein calories to reduce the risk of liver complications related to TPN. Consequently, modern PN solutions are based on a mixture of dextrose and intravenous lipid emulsion to provide the correct balance between calories provided by carbohydrates and by lipids and to prevent essential fatty acid deficiency (EFAD) [61, 62]. In addition, continuous administration of parenteral nutrition results in hyperinsulinemia that predisposes to hepatic steatosis and lipogenesis. Discontinuing TPN infusion for an ideal time of 8 hours every day results in lower insulin levels and reduction of the serum level of transaminases [63].

Lipid emulsions provide what have traditionally been known as the 2 essential fatty acids, linoleic acid (LA) and a-linolenic acid (ALA). These fatty acids are important precursors of eicosanoids and prostaglandins [64] and are imperative essentials in many biochemical pathways. Lipid emulsions derived from soybean oils are the standard of care in the United States and have been shown to cause liver injury both in vitro and in vivo in rodent models [65–67]. This is most likely due to the high concentration of omega-6 fatty acids and phytosterols, which are associated with impaired biliary secretion [68]. Outside the United States, several alternatives are available, including Lipofundin (50% soybean oil and 50% coconut oil) and ClinOleic (20% soybean oil and 80% oleic oil).

Fish oil contains docosahexaenoic acid (DHA) and eicosapentaenoic acid (EPA) and has anti-inflammatory properties due to the inhibition of the arachidonic acid (AA) pathway, leukotrienes, prostaglandins and thromboxane A3 [69–74]. A recent review of 42 patients enrolled within the open-label trial of Omegaven, a fatty acid emulsion rich in omega-3 fatty acids refined from fish oil, showed that patients who received the fish oil-based lipid emulsion experienced reversal of cholestasis approximately 6 times faster (95% confidence interval, 2.0–37.3) than those receiving a soybean oil-based lipid emulsion [75]. The reversal of cholestasis allowed for eventual discontinuation of PN and subsequent development of enteral tolerance. Fish oil monotherapy has additionally been shown to result in significant improvement in all major serum lipid panels although it is not available in the USA [76].

### 14.3. Pharmacological Therapy

Ursodeoxycholic acid (UDCA) is a hydrophilic bile acid that inhibits ileal absorption of toxic bile salts and has cytoprotective, antiapoptotic, antioxidant, and immunomodulatory properties [77]. Additionally, UDCA stimulates the expression and function of hepatobiliary transport systems in normal hepatocytes [78]. There have been several studies that have shown that patients receiving long-term PN at a dosage of 15–30 mg·kg^−1^ per day had lower serum level of liver enzymes although the long-term benefits are still unknown [79–82].

Metronidazole has also been used to prevent steatosis in obese patients who underwent jejunoileal bypass surgery [83]. One of the mechanisms that metronidazole might be beneficial in patients with SBS is that it inhibits bacterial overgrowth and the intestinal deconjugation of bile acids. Lambert and Thomas [84] showed that metronidazole prevented elevated serum liver enzymes in patients receiving PN. Similarly, another group reported the benefits of metronidazole on liver function tests in patients with Crohn's disease requiring PN [85]. Despite these promising findings, the outcomes of long-term use of metronidazole in patients with SBS are still not completely known.

## 15. Surgical Therapy

### 15.1. Prophylactic Cholecystectomy

Complications from cholelithiasis are more frequent in SBS in both adult and pediatric patients compared to the general population. Therefore, prophylactic cholecystectomy is recommended in SBS patients when laparotomy is being undertaken for other reasons [53, 54].

### 15.2. Bianchi Procedure and Serial Transverse Enteroplasty (STEP)

Two surgical techniques have been used to improve the intestinal function of patients with intestinal insufficiency. The first procedure was described by Bianchi in 1980 [86] where the bowel is divided longitudinally and each half is remodeled into a tube with a smaller lumen (one half the original diameter). The two lengths of bowel are subsequently anastomosed in an end-to-end fashion with the final result of doubling of the overall length and surface area of the intestine [87].

The second procedure was described by Kim et al. in 2003 and it is named serial transverse enteroplasty procedure (STEP) [88]. When performing STEP, the bowel is serially stapled at equal intervals so that the bowel takes a zig-zag configuration that increases the transient time of the intestinal content resulting in better absorption of nutrients. Both operations are associated with 81–89% survival rates, 47–54% weaning from PN, and 82–85% overall improvement of intestinal function [87].

### 15.3. Intestinal Transplantation

Small bowel transplant is considered the only definitive treatment for patients with SBS who failed intestinal rehabilitation. In general, patients with less than 50 cm of small bowel and without colon should be referred to a transplant center as soon as possible as the majority will depend on PN for their survival [36]. In the past, indications for small bowel transplantation were restricted only to patients with life-threatening complications due to intestinal failure (Table 5) [89, 90]. In recent years, the experience accumulated has shown that outcomes are significantly improved and small bowel transplant can prevent and often reverse noncirrhotic liver disease caused by long-term PN [91].

Therefore, when considering intestinal transplantation, it is important to determine whether the recipient is affected by irreversible liver disease. In the presence of a liver biopsy showing the presence of fibrosis or cirrhosis, the patient should be considered for simultaneous liver and small bowel transplantation that provide survival rates at 1 year of 90%. Regardless of the type of transplant required, early referral is recommended as some of the complications caused by long-term PN can be prevented by the expertise provided by multidisciplinary teams available at transplant centers.

## 16. Summary

IFALD is a common and potentially life-threatening condition for patients with SBS requiring long-term PN. There exists the potential for decreasing its incidence by optimizing the composition and the rate of infusion of parenteral solutions, by advocating a multidisciplinary approach, and by early referral for intestinal-liver transplantation to ensure long-term survival of patients with SBS.

## Figures and Tables

**Table 1:** The most common causes of short bowel syndrome in 300 adults treated at the University of Nebraska Medical Center from 1990 to 2005.

Main causes of short bowel syndrome	Number	%
Postoperative resection	84	28
Malignancy/irradiation	63	21
Mesenteric vascular disease	63	21
Crohn's disease	49	16
Trauma	22	8
Other benign conditions	19	6

**Table 2:** The spectrum of hepatobiliary complications in patients with short bowel syndrome receiving long-term parenteral nutrition.

Hepatobiliary complications	
Abnormal liver function tests	
Steatosis	
Cholestasis	
Fibrosis	
Cirrhosis and hepatocellular carcinoma	
Liver failure	
Choleltihiasis, cholecystitis, and acalculous cholecystitis	
Biliary sludge	

**Table 3:** Etiology of liver disease in SBS patients receiving prolonged parenteral nutrition.

Etiological factors	
Duration of parenteral nutrition	
Length of bowel remnant	
Compromised enterohepatic circulation	
Lack of enteral nutrition	
Recurrent episodes of sepsis	
Toxic components of parenteral nutrition such as peroxides	
Protein deficiency	
Lack of essential fatty acids and chlorine	
Excess of dextrose and/or lipids	

**Table 4:** Histopathological findings and pathophysiology of intestinal failure associated liver disease (IFALD).

IFALD stage	Events	Mediators/factors	Parenchymal changes	References
Early stage	Steatosis: abnormal accumulation of lipids in hepatocytes	Tumor necrosis factor *α* (TNF-*α*) Fas ligands (Fas-L)	Activation of caspase pathway that leads to cell damage	[38–41]

Intermediate stage	Steatohepatitis: release of reactive oxygen radicals and other proinflammatory mediators causing cellular membrane dysfunction and increased mitochondrial permeability	Oxydative damage of cellular and membrane lipids	Inflammation involving hepatic and perihepatic cells (steatohepatitis) Cellular dysfunction causing cholestasis	[42–44]

Advanced stage	Hepatocytes ballooning, bile duct proliferation, and cirrhosis	Oxygen radicals and proinflammatory mediators causing hepatocyte death or apoptosis	Irreversible scarring of the liver tissue (cirrhosis)	[42–46]

**Table 5:** Established indications for intestinal transplantation in patients on parenteral nutrition.

Failure of parenteral nutrition	
Thrombosis of ≥2 central veins with inability to provide parenteral nutrition	
At least 2 episodes per year of severe bacterial sepsis or fungemia	
Liver failure secondary to long-term parenteral nutrition	
